# An integrative algorithm combining HLA epitope registry, PIRCHE-T2, and PIRCHE-B outcomes to improve immunological risk stratification in kidney transplantation

**DOI:** 10.3389/fimmu.2025.1718506

**Published:** 2026-01-16

**Authors:** He Zhao, Pramath Kakodkar, Eric Wang, Dan Zhang, Matthias Niemann, Destinie Webster, Twyla Pearce, Ahmed Shoker, Paul Keown, Karen Sherwood, Fang Wu, Cody Lewis, Ahmed Mostafa

**Affiliations:** 1 Department of Obstetrics and Gynecology, Beijing Anzhen Hospital Affiliated to Capital Medical University, Beijing, China; 2 Histocompatibility and Immunogenetics Laboratory, St. Paul’s Hospital, Saskatoon, SK, Canada; 3 College of Graduate and Postdoctoral Studies, University of Saskatchewan, Saskatoon, SK, Canada; 4 Department of Pathology and Laboratory Medicine, University of Saskatchewan, Saskatoon, SK, Canada; 5 College of Medicine, University of Saskatchewan, Saskatoon, SK, Canada; 6 PIRCHE AG, Research and Development, Berlin, Germany; 7 Saskatchewan Transplant Program, Saskatchewan Health Authority, Saskatoon, SK, Canada; 8 Vancouver Coastal Health, University of British Columbia, Vancouver, BC, Canada; 9 Department of Pathology, Brigham and Women’s Hospital, Boston, MA, United States; 10 Harvard Medical School, Boston, MA, United States

**Keywords:** epitope, HLA, immunological risk, kidney transplantation, molecular mismatch, PIRCHE, Snow

## Abstract

**Aim:**

Kidney transplantation remains the most effective treatment for end-stage kidney disease. Still, the development of *de novo* donor-specific antibodies (dnDSA) increases the risk of rejection and allograft failure. While molecular matching algorithms assess B-cell and T-cell epitope mismatches, no single method fully captures rejection risk across immune pathways. This study combines the HLA Epitope Registry (Epregistry), PIRCHE-T2, and PIRCHE-B scores to enhance risk stratification, allowing for early intervention in high-risk recipients and improving long-term outcomes.

**Methods:**

A retrospective study of 594 kidney transplant recipients in Saskatchewan (1981–2021), Canada, was conducted, tracking *de novo* donor-specific antibodies (dnDSA) development until January 2024. Epitope mismatch scores were calculated using Epregistry, PIRCHE-T2, and PIRCHE-B, and receiver operating characteristic (ROC) curve analysis determined the optimal cutoff values for predicting dnDSA formation. Patients were categorized into high-risk (all scores > cutoff), intermediate-risk (one algorithm > cutoff), and low-risk (all scores < cutoff) groups. Kaplan-Meier survival analysis evaluated dnDSA-free survival across risk categories.

**Results:**

Among 594 recipients, 104 individuals (17.5%) developed *de novo* DSA; of these, 29 patients developed more than one, resulting in a total of 146 dnDSA events. The most frequently targeted locus was HLA-DQ (72/146, 49.3%), followed by HLA-DR (25/146, 17.1%) and HLA-A (24/146, 16.4%). The optimal cutoff values for predicting dnDSA were 22.5 (Epregistry), 30.5 (PIRCHE-T2), and 5.5 (PIRCHE-B) for Class I, and 15.5 (Epregistry), 17.5 (PIRCHE-T2), and 5.5 (PIRCHE-B) for Class II (all p < 0.05). Across all molecular mismatch load metrics, Kaplan–Meier analysis demonstrated significantly lower dnDSA-free and antibody-mediated rejection (ABMR)-free survival among high-risk recipients compared with low-risk recipients (log-rank p < 0.001). In addition, both the PIRCHE-T2 score at HLA Class I loci and the overall PIRCHE-T2 score were significantly associated with T-cell mediated rejection (TCMR) (p < 0.01).

**Conclusion:**

Integrating Epregistry, PIRCHE-T2, and PIRCHE-B enhances risk stratification for kidney transplant recipients. Epregistry and PIRCHE-B evaluate HLA antibody epitope mismatches, and PIRCHE-T2 focuses on T-cell mismatches. Applied in conjunction, the methods show improved predictive accuracy, making this multi-algorithm approach more effective in identifying high-risk patients. By enabling earlier interventions and personalized immunosuppressive strategies, this model has the potential to improve long-term transplant success.

## 1 Introduction

Solid organ transplantation is the definitive treatment for end-stage organ failure. However, renal allograft rejection is a major contributor to transplant failure, with a reported incidence of 21-22% (1). Donor-specific antibodies (DSA) targeting human leukocyte antigens (HLA) are the main drivers of antibody-mediated rejection (ABMR). The development of *de novo* DSA (dnDSA) after transplantation significantly compromises the graft survival and increases the risk of both antibody-mediated rejection (ABMR) and T cell-mediated rejection (TCMR) (2, 3).

HLA matching is a fundamental strategy for minimizing alloimmune responses in transplantation. Over time, the concept of HLA matching has evolved from the macroscopic antigen level to the microscopic molecular level. This evolution is based on the understanding that HLA antibodies and B cell receptors recognize discrete antigenic sites (epitopes) on the HLA molecules rather than the entirety of the HLA molecule, and that epitope-level matching may provide a more rational framework for assessing alloimmune risk in transplantation (4). Several B-cell epitope mismatch algorithms have been developed by analyzing the differences in the amino acid composition, structure, or physicochemical properties of donor HLA proteins. HLA Matchmaker was the first computational tool developed to assess donor–recipient compatibility at the molecular level by analyzing mismatches based on HLA eplets (5). Building on this concept, the International Registry of HLA Epitopes (Epregistry) (http://www.epregistry.com.br) was established in 2013 as a publicly accessible and continuously updated database. It catalogs eplets and antibody-verified epitopes and provides online tools, such as the HLA Eplet Mismatch Calculator, to quantify mismatches between donor and recipient alleles. Epregistry has since become an essential resource for investigating immune responses to HLA mismatches in transplant recipients and exploring histocompatibility at the epitope level (6).

In addition to HLAMatchmaker and other eplet-based approaches, novel algorithms have now leveraged structural modeling to refine their B-cell epitope mismatch prediction. One such tool is Snow, which integrates the Snowflake algorithm that calculates the solvent-accessible surface area of individual amino acids in HLA proteins, with the Snowball algorithm, which identifies the local surface protrusions of amino acids. The Snow algorithm counts the number of mismatched donor-HLA-specific, exposed amino acid positions, referred to as the PIRCHE-B score, and thus provides a systematic approach to predicting B-cell epitope mismatches in transplantation (7, 8).

While B-cell epitope mismatch algorithms primarily focus on predicting antibody targets related to ABMR, T cell-mediated alloimmune responses are also key modulators of graft rejection. To capture this dimension, the algorithm originally introduced as PIRCHE-II (Predicted Indirectly Recognizable HLA Epitopes) was later standardized as PIRCHE-T2. It predicts the number of potential donor-derived peptides that can be presented by the recipient’s HLA class II molecules and participate in indirect CD4^+^ T-cell allorecognition, reported as the PIRCHE-T2 score in this study. Numerous studies have demonstrated that lower PIRCHE-T2 scores are associated with better outcomes following solid organ transplants (such as heart, lung, and liver transplants) (9–11).

Due to the interplay between B- and T-cells in allograft rejection, integrated epitope mismatch assessment is likely to provide a more accurate immunological risk stratification than evaluating either pathway in isolation (12, 13). Building on this concept, we propose a combinatory and complementary algorithm to further enhance the predictive accuracy over the classical single-pathway approaches.

## 2 Methods

### 2.1 Study design

This single-center retrospective study was conducted with the approval of the Biomedical Research Ethics Board (Bio-REB4110) at the University of Saskatchewan, Saskatoon, Canada. Patient-level clinical data were retrieved from the provincial electronic health records system following ethics approval. Of 615 kidney transplant recipients initially identified, 594 had complete data and were included in the final analysis. These individuals underwent kidney transplantation in Saskatchewan between January 1981 and December 2021 and were followed from the time of transplantation until either biopsy-confirmed rejection or January 1, 2024, whichever came first. The earliest occurrence of dnDSA during follow-up was also recorded. Graft loss and death were not analyzed as outcomes in this study, and no deaths occurred in the cohort.

Deceased donor kidney allocations followed the provincial Saskatchewan donor allocation system or the Canadian Transplant Registry (CTR) for out-of-province donors. Living donor transplants followed standard live donation protocols. Clinical and demographic data, including age, sex, ethnicity, ABO blood type, pregnancy, transfusion, and transplant history, dnDSA characteristics, and protocol biopsy findings, were collected for analysis.

### 2.2 DNA extraction and HLA typing

DNA was extracted from whole blood collected in EDTA tubes using the QIAGEN EZ1^®^ Advanced XL or the QIAGEN EZ2^®^ Connect (Qiagen, Toronto, Canada) and the EZ1&2™ DNA Blood 350 μl kit (Catalog 951054). DNA concentration and purity were assessed with a NanoDrop One spectrophotometer (Thermo Fisher Scientific, USA), and only samples with a 260/280 ratio between 1.7 and 2.0 were processed. HLA genotyping was then carried out using One Lambda’s AllType™ FASTplex NGS 11 Loci Flex kits (One Lambda, Thermo Fisher Scientific, USA) on the Illumina MiniSeq platform (Catalog FC-420-1004, Illumina, USA), covering HLA loci A, B, C, DRB1, DRB3/4/5, DQA1, DQB1, DPA1, and DPB1. Data were analyzed using TypeStream Visual (TSV) version 3.0 NGS software (One Lambda, Thermo Fisher Scientific, USA) (14). For patients previously HLA typed by CDC or SSO (One Lambda, Thermo Fisher Scientific, USA), and for whom DNA is not available for NGS re-typing, high-resolution allele predictions were generated using the HaploStats tool (https://www.haplostats.org/haplostats). HaploStats estimates the most likely high-resolution alleles based on haplotype frequencies and self-reported ethnicity, referencing the National Marrow Donor Program (NMDP) 2011 dataset. In addition to HaploStats, high-resolution imputation from low-resolution typings was also performed using the PIRCHE-T2 and PIRCHE-B algorithms. Both platforms are validated for imputation from low-resolution data and use population haplotype frequencies to resolve the most probable high-resolution genotypes (15). These algorithms have been used in multiple transplant studies involving imputed HLA data, including prediction of donor-specific antibody formation and molecular eplet mismatch assessment.

In this study, haplotype/allele assignments were accepted when the top-ranked solution showed clear posterior probability dominance, defined as either being at least twofold higher than the next most likely assignment or achieving a cumulative probability of approximately 80–90% when multiple equivalent solutions were reported. When no dominant assignment was identified, imputed alleles were further confirmed by concordance across HaploStats, PIRCHE-T2, and Snow., as well as by comparison with allele frequencies observed in an internal Saskatchewan high-resolution reference dataset (>3,000 individuals). This integrative validation strategy demonstrated strong inter-method agreement, with over 95% of imputed alleles aligning with locally observed allele frequencies, supporting the robustness of our imputation approach despite the absence of a predefined HaploStats cutoff.

### 2.3 HLA antibodies

All patients underwent at least annual testing, with more frequent assessments if clinically indicated. *de novo* DSA was strictly defined as donor-specific antibodies that were newly detected at least 3 months post-transplantation, in patients who had no detectable DSA at the time of transplantation and in all subsequent tests up to that point. Anti-HLA antibody detection, including donor-specific antibody (DSA) identification, was performed using single-antigen bead (SAB) assays in our American Society for Histocompatibility and Immunogenetics (ASHI) accredited histocompatibility laboratory, following the manufacturer’s instructions (One Lambda, Thermo Fisher Scientific, USA). All serum samples were treated with a 0.5 M EDTA disodium salt stock solution (Sigma-Aldrich, E7889; St Louis, MO) to a final concentration of 6.2 mM prior to testing. Patient serum (20 µL) was incubated with fluorescently coded microbeads, each coated with a unique purified HLA antigen. After washing to remove unbound antibodies, PE-conjugated anti-human IgG was added, followed by a second incubation. All samples were analyzed on either the Luminex FLEXMAP 3D platform or the Luminex 200 system. A divisor of 1.67 was applied during data analysis to adjust for systematic differences in mean fluorescence intensity (MFI) measurements between the two instruments, and results were analyzed using HLA Fusion software version 4.4 (One Lambda, Thermo Fisher Scientific, USA). Antibody reactivity was quantified using trimmed median fluorescence intensity (MFI), with positivity determined by comparison to internal negative controls. An MFI threshold of ≥1000 was used to define clinically relevant reactivity. This cutoff was adopted based on previously validated thresholds in the literature and our institutional protocol, including prior findings demonstrating its association with increased risk of antibody-mediated rejection and inferior allograft outcomes, particularly in living donor settings. DSAs were defined as positive SAB reactivity against donor-specific HLA antigens exceeding this 1000 MFI threshold. This approach is consistent with recent evidence, including our prior work (16, 17), supporting its clinical utility for immunologic risk stratification in kidney transplantation. dnDQA1-DSA was excluded from the analysis.

### 2.4 HLA epitope mismatch assessment

Epitope mismatches between donors and recipients for HLA-A, -B, -C, -DRB1/3/4/5, and -DQA1/DQB1 loci were assessed using the Epregistry tool (v2024-08-19; http://www.epregistry.com.br), a public resource for calculating epitope mismatch scores. PIRCHE-B and PIRCHE-T2 molecular mismatch load scores at HLA-A, -B, -C, -DRB1, and -DQB1 loci were determined using Snow (v4.3; Snow 1.1, surface area: 0.26, protrusion: 0.68, IMGT 3.54) and PIRCHE-T2 (v4.2; Frost 1.1, binding rank: 30%, IMGT 3.54) via the commercial TxPredictor platform at (http://www.pirche.com). Total epitope mismatch scores from each tool were categorized by HLA Class (I: A/B/C; II: DR/DQ) and individual loci. HLA-DP epitope scores were excluded from analyses due to incomplete DP typing across the cohort. A comparative summary of the underlying principles, immunologic scope, and key characteristics of these three distinct algorithms (Epregistry, PIRCHE-T2, and PIRCHE-B is provided in **Supplementary Table 1**. Complete matched pairs were excluded. Only verified eplets listed in the Epregistry database were included in the analysis.

### 2.5 Immunosuppression and postoperative management

All transplanted patients received induction therapy following institutional policy, which includes either basiliximab or anti-thymocyte globulin (ATG). For maintenance immunosuppression, patients were prescribed a standard triple-drug regimen consisting of a calcineurin inhibitor (tacrolimus, sirolimus, or cyclosporine), an antiproliferative agent (mycophenolate mofetil or azathioprine), and corticosteroids, tailored to individual clinical needs. ABO incompatibility kidney transplant was not performed.

All transplant follow-up is performed at one center. Biopsies are performed for unexplained increases in serum creatinine by 20 percent or suspected rejection due to adherence issues. Testing for DSA is routinely performed at the time of biopsy. Allograft rejection was diagnosed on for-cause biopsies performed in response to clinical indications such as graft dysfunction, increased creatinine, or proteinuria. All biopsies were reviewed by renal pathologists at the Saskatchewan Health Authority (SHA) in Saskatoon, Saskatchewan, and classified according to the Banff Kidney Allograft Classification, applying the criteria in effect at the time of diagnosis (1997–2022). Histopathologic evaluation included assessment of interstitial inflammation (i), tubulitis (t), arteritis (v), glomerulitis (g), peritubular capillaritis (ptc), and C4d deposition, among other relevant parameters.

### 2.6 Statistics

Categorical variables were summarized as frequencies and compared between groups using the chi-square test. Non-normally distributed variables (e.g., HLA epitope mismatch scores) were reported as median with interquartile range (IQR) and compared using the Mann-Whitney U test. To correct for multiple testing, p-values were adjusted by the Benjamini–Hochberg method, with a false discovery rate (FDR) < 0.05 considered statistically significant.

Thresholds of epitope mismatch scores predictive of dnDSA development were determined by receiver operating characteristic (ROC) curve analysis, and optimal cutoffs were defined using the Youden index. Kaplan–Meier analysis was applied to estimate the cumulative incidence of dnDSA and ABMR, with differences assessed by the log-rank test. The association between ABMR and DSA was evaluated using the Phi coefficient for binary variables. Multivariable Cox proportional hazards regression analyses were performed to evaluate the association between molecular mismatch risk classification and time-to-event outcomes, adjusting for recipient age, ethnicity, and donor type.

Internal validation of the integrative algorithm was performed using two complementary approaches. First, split-sample cross-validation was conducted within SPSS, in which the dataset was randomly divided into training and validation subsets, and the area under the ROC curve (AUC) was computed for both. Second, bootstrap resampling with 1,000 iterations was applied to the logistic regression models using the SPSS bootstrap module. For each comparison (intermediate vs. low risk, high vs. low risk), bias-corrected and accelerated (BCa) 95% confidence intervals were generated to obtain robust estimates of regression coefficients and odds ratios.

All statistical analyses were performed with SPSS for Mac (version 29.0.1.0; IBM Corp., Armonk, NY, USA) and GraphPad Prism for Mac (version 10.0.0; GraphPad Software, Boston, MA, USA).

## 3 Result

### 3.1 Baseline characteristics of the cohort

Of the 594 patients who received a kidney transplant, 104 developed dnDSA (**Table 1**). Recipients who developed dnDSA were younger than those who did not (51.0 (27) vs. 57.0 (22) years, *p = 0.009*); however, the small effect size indicates limited clinical impact (Cohen’s d = -0.21). The sex distribution was similar between groups (54.8% vs. 61.2% male, *p = 0.225*), as were rates of prior transplantation (4.8% vs. 5.3%, *p = 0.836*), pregnancy (15.4% vs. 10.2%, *p = 0.127*), and blood transfusion (22.1% vs. 20.4%, *p = 0.696*). Among the 494 recipients with available ethnicity data, a significant difference in composition was observed (*p = 0.012*), with the positive dnDSA group (dnDSA^+^) comprising a higher percentage of First Nations [29.9% (23/77)] compared to Caucasian [15.5% (57/367)] or other [20.0% (10/50)]. ABO blood group distribution did not differ significantly between groups (*p* = 0.696). Notably, the dnDSA^+^ group had a shorter mean follow-up time (81.5 (107) vs. 102.1 (143) months, *p* = 0.031), possibly reflecting earlier post-transplant immunologic events. However, no significant difference was seen in ABMR-free survival time (322.0 (409) vs. 239.0 (352) months, *p = 0.141*). Donor characteristics, including age (52.4 ± 14.8 vs. 50.7 ± 15.6 years, *p = 0.367*) and sex (47.7% vs. 49.5% female, *p = 0.762*), were comparable between groups. Living donor rates were also similar (38.5% vs. 32.4%, *p = 0.238*).

**Table 1:** Baseline characteristics of 594 kidney transplant recipients.

N=594	dnDSA+ (n=104)	dnDSA- (n=490)	*P* value
Recipient			
Age [median, IQR] years	51.0 (27)	57.0 (22)	0.009^*^
Sex			
Male n (%)	57 (54.8)	300 (61.2)	
Female n (%)	47 (45.2)	190 (38.8)	0.225
Re-transplant n (%)	5 (4.8)	26 (5.3)	0.836
Pregnancy	16 (15.4)	50 (10.2)	0.127
Blood transfusion	23 (22.1)	100 (20.4)	0.696
Ethnicity			
Caucasian (n=367) n (%)	57 (15.5)	310 (84.5)	
First nation (n=77) n (%)	23 (29.9)	54 (70.1)	
Others (n=50) n (%)	10 (20.0)	40 (80.0)	0.012^*^
Blood type^b^			
A (n=258)	46 (17.8)	212 (82.2)	
B (n=54)	7 (13.0)	47 (87.0)	
O (n=245)	45 (18.4)	200 (81.6)	
AB (n=25)	3 (12.0)	22 (88.0)	0.696
Follow-up time [median, IQR] months	81.5 (107)	102.1 (143)	0.031^*^
ABMR-free time [median, IQR] months	322.0 (409)	239.0 (352)	0.141
Donor			
Age	53.0 (25)	52.5 (22)	0.458
Sex^c^			
Male (n=252)	46 (52.3)	206 (50.5)	
Female (n=244)	42 (47.7)	202 (49.5)	0.762
Living donor	40 (38.5)	159 (32.4)	0.238

*P<0.05.

**Figure 1:**
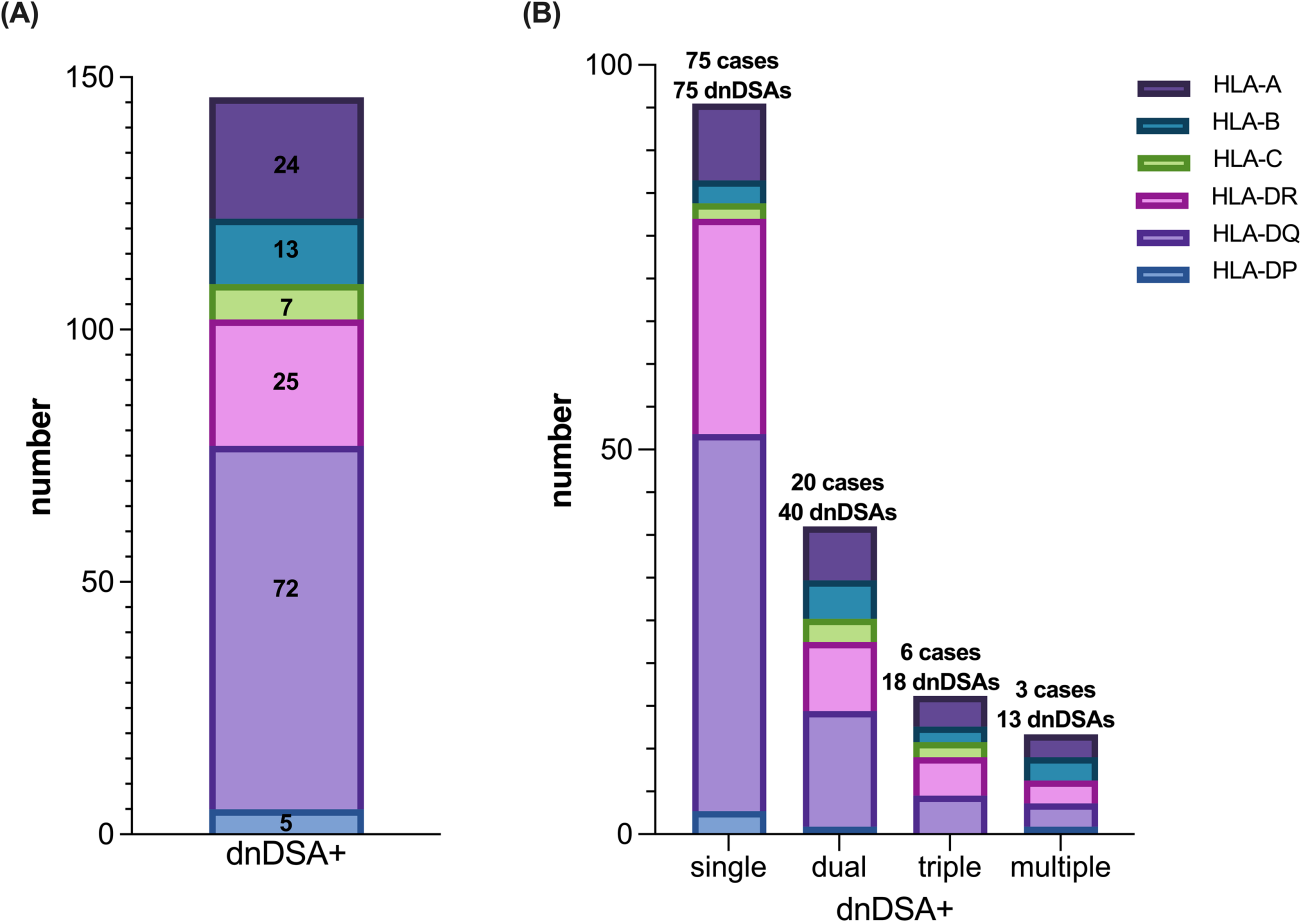
Most dnDSA targeted HLA-DQ. Proportion and types of dnDSA in the total patient population **(A)** and distribution of single versus multiple antibody types among 146 occurrences **(B)**.

Among 594 recipients, 104 individuals (17.5%) developed *de novo* DSA during the follow-up period, including 29 who had multiple antibodies, yielding a total of 146 dnDSA events. The majority of dnDSAs were directed against the HLA-DQB1 locus, occurring in 72 patients (49.3%), highlighting the prominent immunogenicity of the DQ antigen in the post-transplant setting (**Figure 1A**). DR- and A-locus dnDSAs were the next most frequent, identified in 25 (17.1%) and 24 (16.4%) cases, respectively. Because multiple antibodies were identified in some individuals, this indicates that a subset of patients developed complex alloimmune responses: 20 patients produced two distinct dnDSAs, six developed three, and three patients had more than three dnDSAs concurrently (**Figure 1B**).

### 3.2 The relationship between molecular mismatch scores and dnDSA

**Figure 2:**
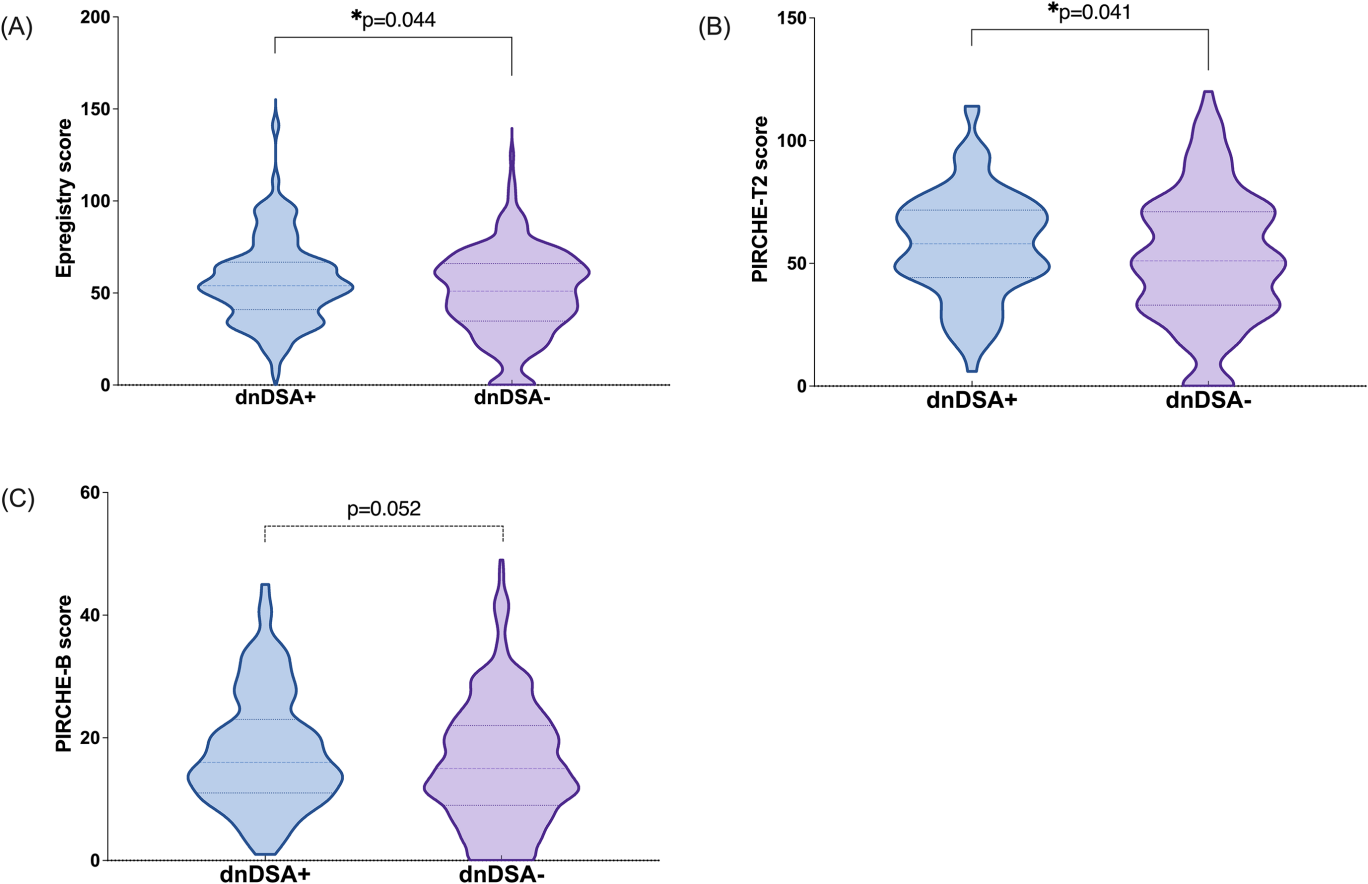
Difference in algorithm scores between dnDSA+ and dnDSA- patients. Epregistry score **(A)**, PIRCHE-T2 score **(B)**, and PIRCHE-B score **(C)** in dnDSA- and dnDSA+ patients. Box and Whisker plots represent the median (line in the middle of the box), 1st and 3rd quartiles (box), and 1.5x interquartile range (whisker). Outliers are depicted as dots outside the whiskers. *p < 0.05.

**Figure 3:**
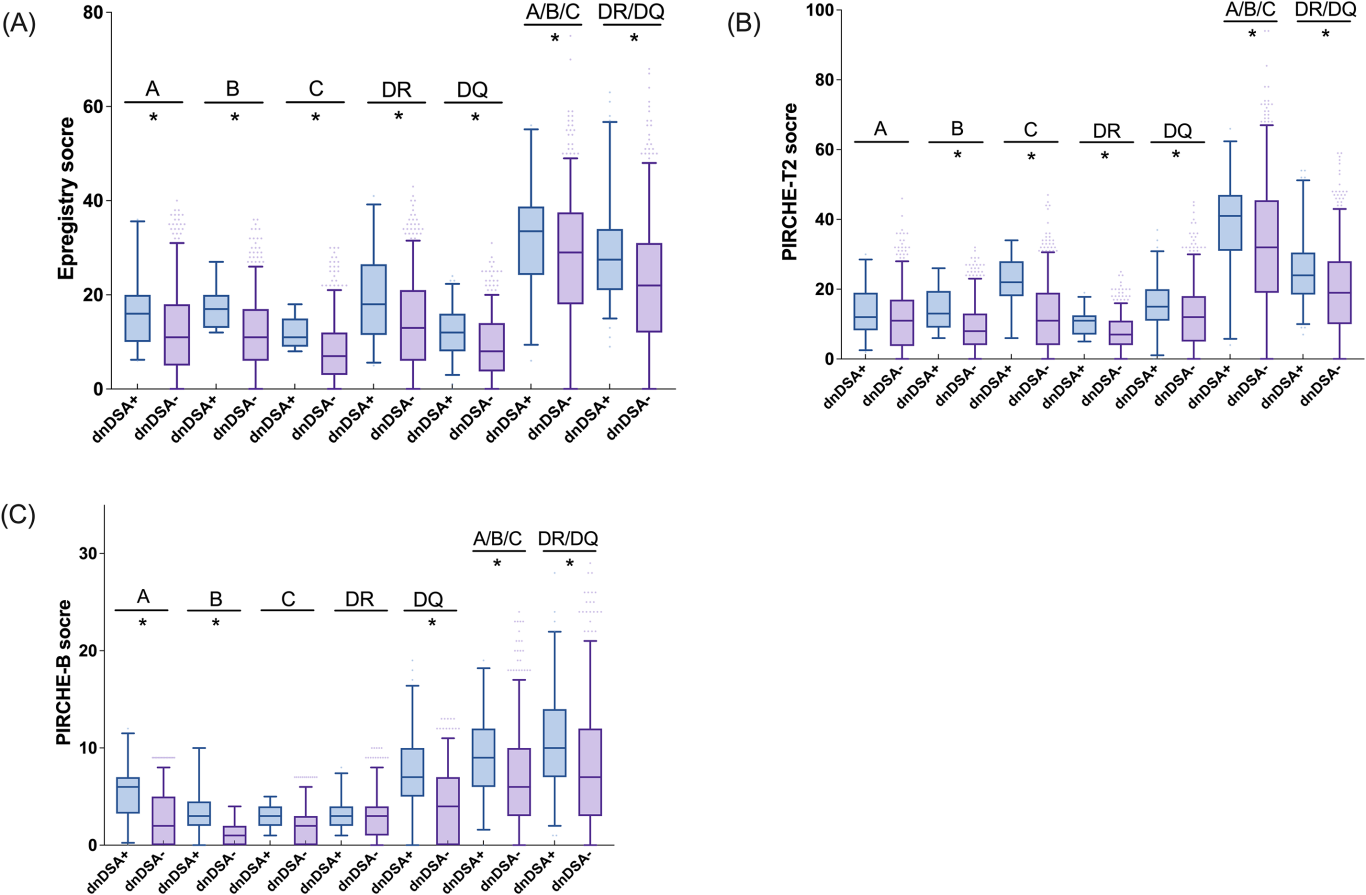
Difference in algorithm scores between dnDSA+ and dnDSA- patients at representative loci. Epregistry score **(A)**, PIRCHE-T2 score **(B)**, and PIRCHE-B score **(C)** in different dnDSA status (positive/negative) at representative loci. Box and Whisker plots represent the median (line in the middle of the box), 1st and 3rd quartiles (box), and 1.5x interquartile range (whisker). Outliers are depicted as dots outside the whiskers. *p < 0.05. All multiple comparisons were adjusted using the Benjamini-Hochberg method to control the false discovery rate (FDR).

The relationship between HLA molecular mismatch burden and dnDSA formation was assessed using Epregistry, PIRCHE-T2, and PIRCHE-B. Epregistry and PIRCHE-T2 showed significantly higher mismatch scores in the dnDSA^+^ group compared to dnDSA^-^ (**Figures 2A, B**), while PIRCHE-B showed a similar trend that narrowly missed significance (**Figure 2C**, p *= 0.052*). Locus- and class-specific analyses revealed distinct mismatch patterns associated with dnDSA status (defined as antibodies targeting the corresponding HLA locus under analysis) (**Figure 3**).

Epregistry mismatch scores were significantly higher in the dnDSA^+^ group across all loci and Classes (**Figure 3A**, p *< 0.05*), demonstrating broad discriminatory power. PIRCHE-T2 scores mirrored these findings, with significant differences observed at HLA-B, -C, -DR, and -DQ, as well as for the combined Class I and Class II scores (**Figure 3B**, p *< 0.05*). Interestingly, although HLA-A scores trended higher in the dnDSA^+^ group, they did not reach statistical significance, suggesting potential variability in the peptide presentation landscape specific to that locus. The PIRCHE-B algorithm showed a similarly robust performance, identifying significant differences at HLA-A, -B, and –DQB1 loci, and across both class levels (**Figure 3C**, p *< 0.05*). Although PIRCHE-B did not show significance at HLA-C and -DR individually, its class-level predictions effectively differentiated dnDSA^+^ from dnDSA^-^ recipients. Across algorithms, consistent Class II signals, particularly at HLA-DQ, underscore the central role of Class II mismatches in post-transplant humoral alloimmunity and support integrating epitope-based metrics into risk stratification.

### 3.3 Risk stratification criteria are proposed based on molecular mismatch scores

Building on the observed associations between molecular mismatch scores and dnDSA development, we next sought to define clinically meaningful thresholds for risk stratification. ROC curve analyses were conducted to determine the optimal cut-off values for predicting dnDSA formation across the three algorithms (**Table 2**; **Supplementary Figure 1**). For HLA Class I loci, the respective cut-off values were 22.5 (Epregistry), 30.5 (PIRCHE-T2), and 5.5 (PIRCHE-B), while for HLA Class II, the thresholds were 15.5, 17.5, and 5.5, respectively. All cut-off values demonstrated statistically significant predictive ability, with area under the curve (AUC) values ranging from 0.601 to 0.648 (*p < 0.05* for all). Using these cut-offs, we categorized patients into three distinct immunological risk groups. Patients whose scores exceeded the threshold across all three algorithms were classified as the high-risk group, those with scores below all cut-offs were designated as low-risk, and patients meeting only one or two cut-off criteria were categorized as intermediate-risk. This stratification framework enabled a more nuanced risk prediction based on the cumulative epitope mismatch burden and forms the basis for our subsequent outcome analyses (18).

**Table 2:** Cut-off of Epregistry, PIRCHE-T2, PIRCHE-B scores identified by ROC curve analysis (ROC curve shown in **Supplementary Figure 1**) to be associated with Class I and Class II dnDSA formation. The risk stratification criterion was established on this basis.

Loci		Algorithm		
		Epregistry	PIRCHE-T2	PIRCHE-B
HLA Class I	Cut-off	22.5	30.5	5.5
	AUC	0.625	0.601	0.642
	p value	0.003^*^	0.017^*^	0.001^*^
HLA Class II	Cut-off	15.5	17.5	5.5
	AUC	0.648	0.632	0.631
	p value	0.000^*^	0.000^*^	0.000^*^
Risk stratification				
HLA Class I	High risk	≥23	≥31	≥6
	Moderate risk	One of 3-algorithm above cut-off		
	Low risk	<23	<31	<6
HLA Class II	High risk	≥16	≥18	≥6
	Moderate risk	One of 3-algorithm above cut-off		
	Low risk	<16	<18	<6

Subsequently, Internal validation was conducted using split-sample cross-validation and bootstrap resampling (**Supplementary Table 2**). In split-sample validation, the AUC for both HLA class I (A/B/C) and class II (DR/DQ) remained consistent with the original estimates. Bootstrap-validated logistic regression further confirmed the stability of the model coefficients. For HLA class I (A/B/C), the comparison of intermediate versus low risk yielded B = 1.278 (SE = 5.664, 95% CI: -0.108 to 18.408, *p* = 0.066), while high versus low risk showed B = 2.258 (SE = 5.655, 95% CI: 1.160 to 19.273, *p* = 0.004). For HLA class II (DR/DQ), intermediate versus low risk yielded B = 1.745 (SE = 5.802, 95% CI: 0.705 to 19.185, *p* = 0.012), and high versus low risk yielded B = 2.604 (SE = 5.803, 95% CI: 1.585 to 19.997, *p* < 0.001).

### 3.4 Kaplan-Meier survival curve for dnDSA-free probability and ABMR-free survival probability

**Figure 4:**
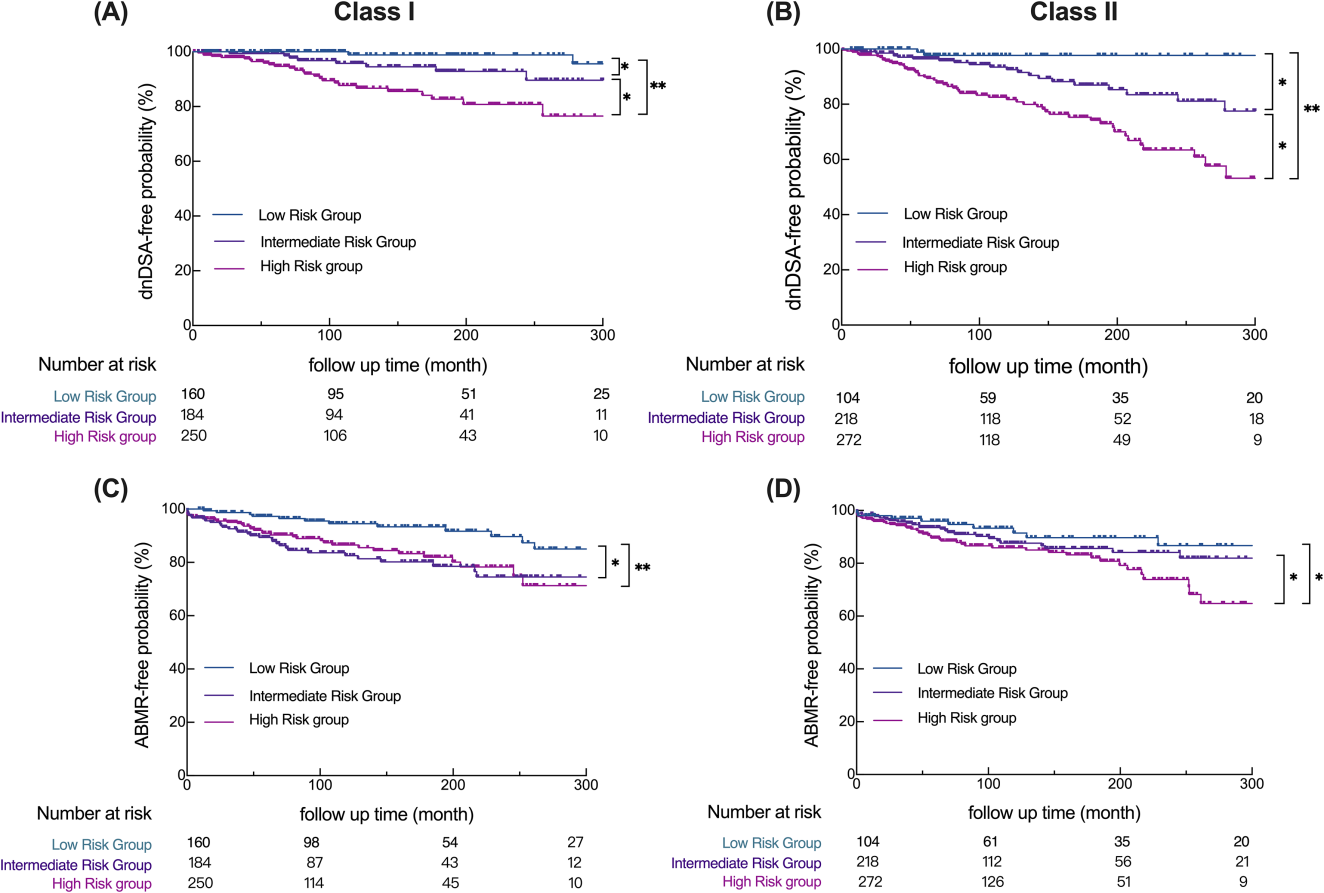
The high-risk group showed a greater propensity for dnDSA development. Kaplan-Meier survival curve for dnDSA-free probability **(A, B)** or ABMR-free probability **(C, D)** in each group based on the risk classification criteria(*P<0.05,**P<0.001).

To evaluate the clinical relevance of the molecular mismatch-based risk categories, we performed Kaplan-Meier survival analyses for dnDSA-free and ABMR-free outcomes across the stratified groups (**Figure 4**). A total of 375 patients underwent pathological biopsy. Among them, 79 were diagnosed with ABMR (48 cases were dnDSA-positive, and 31 cases were dnDSA-negative), and 104 were diagnosed with TCMR, including 39 patients who had both ABMR and TCMR. Of the 104 dnDSA-positive patients, 48 developed ABMR and 56 did not.

Patients categorized as high risk based on surpassing the algorithm-specific cut-off values demonstrated the lowest dnDSA-free survival probability over time in both HLA Class I and Class II analyses (**Figures 4A, B**). In contrast, those in the low-risk group exhibited the highest dnDSA-free survival, with the intermediate-risk group falling between these two extremes. All groupwise comparisons reached statistical significance (*p < 0.05*), indicating robust stratification power of the combined algorithmic thresholds. To account for the limited number of patients at extended follow-up, Kaplan–Meier curves were truncated at 300 months, as 95% of follow-up events occurred by 275.27 months. The full, untruncated survival curves and corresponding data are provided in the Supplementary Materials (**Supplementary Figure 2**).

Consistent patterns were observed in the analysis of ABMR-free survival (**Figures 4C, D**). Again, the high-risk group showed the most pronounced decline in ABMR-free probability, particularly within the first few years post-transplant, while the low-risk group maintained superior outcomes. Statistically significant differences were primarily observed between high- and low-risk groups for both Class I and Class II. To assess whether underexposure or poor adherence to immunosuppression contributed to the observed differences, we compared tacrolimus trough levels between recipients who developed AMR and those who did not. Median trough concentrations were comparable between groups (7.2- 7.3 nmol/L), with no statistically significant difference observed (p > 0.05; **Supplementary Figure 3**). These results suggest that variability in immunosuppressive drug exposure was not a major determinant of dnDSA formation or AMR in this cohort. Rather, the data support the interpretation that high HLA molecular mismatch burden was the primary driver of alloimmune activation. Taken together, these findings highlight the prognostic value of integrated epitope mismatch scoring in predicting dnDSA development and antibody-mediated rejection, independent of baseline immunosuppression levels. Furthermore, the Phi coefficient of 0.446 between dnDSA and ABMR suggests a moderate positive association, supporting the clinical link between immunological sensitization and graft injury (**Supplementary Table 3**). Collectively, these data reinforce the practical applicability of integrated molecular mismatch scoring in risk stratification and outcome prediction in kidney transplantation. In parallel, the incidence of dnDSA, ABMR, and TCMR also increased progressively across the low-, intermediate-, and high-risk groups, reinforcing the prognostic utility of molecular mismatch-based risk stratification (**Supplementary Figure 4**).

Multivariable Cox regression analyses were performed separately for class I and class II dnDSA and ABMR endpoints. Across all four models, molecular mismatch risk classification demonstrated a significant overall association with time-to-event outcomes after adjustment for recipient age, ethnicity, and donor type. Higher molecular mismatch burden was consistently associated with an increased risk of dnDSA development and ABMR, with stronger effect sizes observed for dnDSA endpoints compared with ABMR. Detailed hazard ratios and confidence intervals for each endpoint are provided in **Supplementary Table 4**.

### 3.5 The relationship between molecular mismatch scores and TCMR

**Figure 5:**
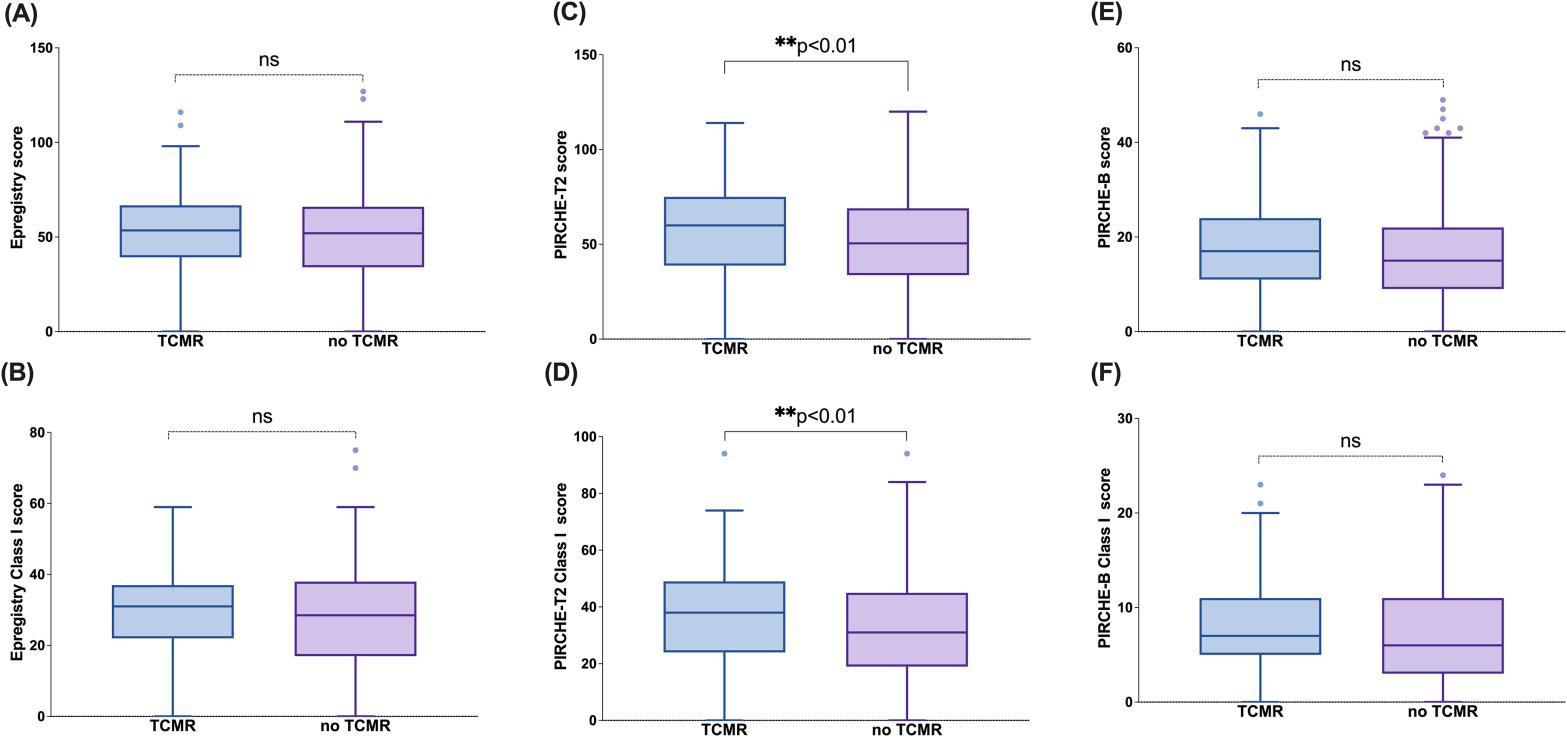
PIRCHE-T2 score is predictive of TCMR. Epregistry score **(A)**, PIRCHE-T2 score **(B)**, PIRCHE-B score **(C)**, Epregistry Class I score **(D)**, PIRCHE-T2 Class I score **(E)**, and PIRCHE-B Class I score **(F)** in different TCMR status (TCMR+/TCMR-) at representative loci. Box and Whisker plots represent the median (line in the middle of the box), 1st and 3rd quartiles (box), and 1.5x interquartile range (whisker). Outliers are depicted as dots outside the whiskers. **p<0.01; ns, not significant.

Following the demonstration of strong associations between molecular mismatch-based risk stratification and dnDSA/ABMR outcomes, we next evaluated whether these algorithms were similarly informative in predicting TCMR. PIRCHE emerged as the only algorithm that demonstrated a statistically significant association with TCMR status, with elevated scores observed in patients who experienced TCMR (**Figure 5**). This was evident in both the overall PIRCHE-T2 score (**Figure 5C**) and the Class I PIRCHE-T2 score (**Figure 5D**), where the TCMR+ group had significantly higher median values compared to the TCMR– group (*p < 0.01*). In contrast, no significant differences were observed between TCMR+ and TCMR– patients for the Epregistry and PIRCHE-B algorithms, either at the total level (**Figures 5A, B**) or for their respective Class I components (**Figures 5E, F**), suggesting limited predictive value of these platforms for TCMR.

Additionally, there was an association between TCMR status and locus-specific scores, including Epregistry-C, PIRCHE-T2-C, PIRCHE-B-C, and PIRCHE-B-B (**Supplementary Figure 5**). While certain locus-level associations reached statistical significance, their clinical utility remains uncertain and requires further, larger studies to confirm.

Taken together, these results suggest that PIRCHE scoring may capture aspects of antigen presentation relevant to cellular rejection, although its predictive performance appears modest and not uniformly replicated across all algorithms or loci.

## 4 Discussion

In this single-center retrospective study of 594 kidney transplant recipients, we evaluated the predictive value of three HLA molecular mismatch scores: Epregistry, PIRCHE-T2, and the newly developed PIRCHE-B (Snow), with the development of dnDSA, ABMR, and TCMR. All three metrics showed significant associations with dnDSA formation, supporting their individual and combined utility in immunologic risk stratification. We therefore propose an integrative strategy that leverages all three algorithms to improve the prediction of dnDSA formation and subsequent graft rejection. Validation analyses confirmed the model’s robustness and indicated clear risk discrimination, with high-risk patients experiencing significantly greater dnDSA incidence than their low-risk counterparts across both HLA class I and class II loci.

Regarding demographic characteristics, we found that age and ethnicity were associated with the development of dnDSA, which is consistent with previous studies (19, 20). Younger recipients are more likely to develop dnDSA due to their robust immune responses and potentially lower compliance with immunosuppression therapy (21). Notably, we observed a high incidence of DSA (29.9%) among kidney transplant recipients of First Nations ethnicity. While this study was not designed to assess the relationship between dnDSA formation and ethnicity, future research can continue to explore the specific underlying reasons, the observed trend highlights the pressing need for further research into systemic inequities in transplantation, including reduced access to living donors, increased comorbidity burden, rare HLA haplotypes that complicate matching, potential barriers to consistent immunosuppressive management, and potential systemic biases in organ allocation (22). Overall, recipient age and ethnicity were the only baseline variables that significantly differed between groups, whereas sensitizing exposures such as prior transplant, pregnancy, or transfusion history did not differ significantly. These findings indicate that traditional sensitization factors were not major drivers of the observed differences in dnDSA development within this cohort.

In the dnDSA-positive cohort, HLA-DQ was the predominant target, with 72 patients (49.32%) developing anti-DQ antibodies (**Figure 1A**). Regardless of the number of antibody types per patient, anti-DQ antibodies consistently represented a large proportion of dnDSA cases (**Figure 1B**). Patients with HLA-DQ dnDSA had a significantly higher incidence of graft rejection and markedly worse death-censored graft survival compared to those without DSA (9, 23, 24). Similarly, in our study, recipients in the dnDSA^+^ group have a higher HLA-DQ mismatch score than those in the dnDSA^-^ group in all 3 evaluated algorithms (**Figure 2**). This dominance of DQ as an immunogenic locus has been well-documented in previous research and was further corroborated in our analysis, where DQ mismatch scores were significantly higher in the dnDSA^+^ group across all three algorithms. These findings reinforce the clinical importance of DQ-focused matching strategies and suggest that current organ allocation practices should be revisited to prioritize minimizing DQ mismatches (25–27).

It is noteworthy that although HLA-B showed high mismatch scores across all three algorithms, its observed dnDSA incidence remained substantially lower than that of HLA-DQ. This discrepancy indicates that molecular mismatch quantity does not directly equate to immunogenic strength and reflects the well-recognized immunodominance of HLA-DQ in humoral alloimmunity. The HLA-DQ heterodimer, composed of two highly polymorphic chains (DQA and DQB), generates a structurally diverse epitope repertoire, and DQ-derived peptides are efficiently processed via the indirect pathway to elicit strong CD4^+^ T-cell help. Consistent with this biology, multiple clinical studies have demonstrated that HLA-DQ mismatch is the strongest independent predictor of dnDSA development. Therefore, despite the quantitatively high mismatch burden at HLA-B, its clinical immunogenicity is markedly lower than that of HLA-DQ, further emphasizing the unique biological and clinical importance of DQ in alloimmune responses.

In evaluating algorithm performance, Epregistry emerged as a strong predictor, demonstrating significant elevation of epitope mismatch scores in dnDSA+ patients at both Class I and Class II loci. This is consistent with its design, which emphasizes structurally accessible eplets likely to elicit antibody responses. PIRCHE-T2, which quantifies peptides derived from donor HLA molecules predicted to be presented by the recipient’s HLA Class II, showed significant associations, particularly at HLA-B, C, DR, and DQ, aligning with its theoretical basis in CD4+ T cell help. Although the overall PIRCHE-B score did not reach significance between dnDSA^+^ and dnDSA− groups, it exhibited similar patterns, with statistically significant differences noted at HLA-A, B, and DQ, suggesting possible refinements, particularly at the DRB1 locus. These differences across loci and algorithms underscore their distinct yet overlapping predictive domains, B-cell-driven humoral immunity captured by Epregistry and PIRCHE-B, as well as T-cell help captured by PIRCHE-T2. At the individual locus and combination loci (Class I means HLA-A/B/C loci, Class II means HLA-DRB/DQB1 loci), Epregistry, which is based on antibody-accessible polymorphic amino acid residues (eplets) present on the HLA of the donor, becomes an indicator to determine HLA epitopes and the humoral alloimmune response, and shows significant elevation in the dnDSA+ group. While PIRCHE-T2 and PIRCHE-B did not display consistent increases across all individual loci (PIRCHE-T2 was elevated at HLA-B, C, DRB, and DQB1; PIRCHE-B at HLA-A, B, and DQB1, **Figure 3**), analysis at the aggregated Class I and Class II levels revealed that all 3 algorithms yielded significantly higher scores in recipients who developed dnDSA. These findings are consistent with recent findings from multiple studies (28, 29), which have reported significant associations between eplet mismatch load and/or PIRCHE-T2 scores with the development of *de novo* DSA or graft outcomes. This concordance arises from the coordinated interplay between B cells and T cells in the adaptive immune response. Following antigen recognition via the B cell receptor (BCR), donor-specific naïve B cells internalize the alloantigen, process it within end lysosomal compartments, and present donor-derived peptides on their surface via the HLA Class II molecules. These antigen-presenting B cells subsequently migrate to the T–B cell interface within secondary lymphoid organs, where they engage CD4^+^ helper T cells bearing cognate T cell receptors (TCRs) specific for the peptide-HLA complexes. Upon engagement, CD4^+^ T cells are activated and, through CD40/CD40L interaction and cytokine secretion, provide the requisite co-stimulatory signals that drive B cell clonal expansion and differentiation into antibody-producing plasma cells (15).

To leverage their complementarity, we developed a composite risk model incorporating all three algorithms. This integrative approach allowed for more refined stratification of patients into low-, intermediate-, and high-risk categories for dnDSA formation (**Figure 4A** and B). These findings highlight the superior ability of the combined use of the three algorithms in identifying patients at high risk for dnDSA, effectively compensating for the limitations of using a single algorithm that focuses only on one part of the alloresponse. After all, high-risk patients identified by a single algorithm were classified into the intermediate-risk group in our study and showed a statistically significant difference from the high-risk group defined in this study in the dnDSA-free probability analysis. Additionally, based on the concordant predictive performance of the three algorithms for dnDSA at the combined loci, we further validated this concept using ABMR, given that dnDSA is a high-risk factor for its development. In this study, the phi-value of dnDSA and ABMR was 0.446, indicating a moderate correlation between the two variables. The ABMR-free probability (**Figures 4C, D**) also shows there was a significant difference between the high-risk group and the low-risk groups, both in Class I and Class II, which further reinforces the importance of combining algorithms for accurately identifying high-risk patients. It is noteworthy that among the 79 patients diagnosed with ABMR, 48 were dnDSA-positive. The remaining 31 patients did not have detectable dnDSA. This occurs because some cases represent early-stage ABMR, where antibody levels are below the threshold of detection. In other patients, biopsy features such as microvascular inflammation and C4d positivity indicate ABMR despite the absence of circulating dnDSA, consistent with seronegative ABMR. This can result from non-HLA antibodies, transient or compartmentalized antibody responses, or technical limitations of current assays (30).

In addition, we observed that the intermediate-risk group in Class I exhibited an ABMR-free survival pattern more closely resembling the high-risk group, whereas in Class II, the intermediate-risk group showed clearer separation between low- and high-risk strata. This divergence may reflect differences in how combinations of high-risk algorithmic scores distribute across Class I and Class II loci, suggesting that the weighting of individual algorithms may not contribute uniformly across loci. Although our study was not specifically designed to dissect the impact of individual algorithmic combinations, these findings highlight the need for future work to further refine how algorithm interactions shape risk stratification, particularly for patients classified as intermediate risk. This direction aligns with our ongoing efforts to evaluate different combinations and integration strategies of the three molecular mismatch algorithms in future studies.

We also investigated the potential of these algorithms to predict TCMR. Given that the PIRCHE-T2 algorithm is designed to identify indirectly recognizable HLA-derived peptides that can be displayed on the recipient’s HLA class II molecules and be recognized by CD4+ T cells (31), we hypothesized that it may have superior predictive ability for TCMR. Therefore, we compared patients with and without TCMR and found that both the overall PIRCHE-T2 score and Class I PIRCHE-T2 scores were significantly different between the two groups (**Figure 5**). This finding supports the effectiveness of PIRCHE-T2 in recognizing T cell epitopes. Although some other algorithms also showed statistically significant differences (**Supplementary Figure 2**), such as Epregistry HLA-C score, PIRCHE-T2 HLA-C score, PIRCHE-B HLA-B score, and PIRCHE-B HLA-C score. We do not currently consider these differences to be of substantial clinical relevance due to the limited sample size.

Despite its strengths, this study has limitations. As a single-center retrospective study, it is subject to selection bias and features a significant cohort size imbalance (dnDSA+ n=104 vs. dnDSA- n=490), which may affect statistical reliability. Furthermore, non-immunologic confounders like medication adherence and comorbidities were not adjusted for due to data unavailability, and future prospective studies should include them. Moreover, the dnDSA outcome was defined as a binary, single-timepoint measure, which fails to capture the dynamic profile of antibody development. Additionally, long-term graft survival data were not available. Future studies should employ larger, more balanced, multi-center cohorts with extended follow-up and serial monitoring to validate these findings.

Additionally, the primary aim of this study was to explore the feasibility of integrating multiple HLA epitope-based algorithms into a unified, interpretable workflow. Therefore, our current findings should be viewed as preliminary. External validation using independent datasets, ideally from other provinces or transplant centers, will be essential to assess the generalizability of our findings across diverse populations and healthcare systems. Specifically, our cohort’s specific ethnic composition (e.g., with a notable representation of First Nations recipients) and its region-specific HLA allele frequencies may limit the global generalizability of our findings. Future validation in more ethnically and geographically diverse populations with varying genetic backgrounds is required to confirm the performance and clinical utility of the integrated algorithm.

An additional limitation of this study is its complete reliance on computational predictions without experimental validation of the epitopes. Since functional immunological methods such as T-cell activation, ELISPOT, or antibody testing were not employed, we could not confirm the actual immunogenicity of the predicted epitopes. The current findings primarily reflect algorithmic risk associations, and future studies are needed to experimentally verify the biological functions of these epitopes to establish their clinical applicability.

The operational complexity and substantial computational costs associated with running three algorithms in parallel challenge their clinical practicality, driving the need for a consolidated solution. Our work underscores the importance of future efforts to develop a unified, comprehensive assessment tool that integrates key predictive factors into a more efficient and cost-effective framework without compromising predictive power.

In conclusion, our study is the first to explore the combination of three existing algorithms to improve the identification of high-risk patients, and this approach was successfully validated in both the dnDSA-positive and negative, ABMR, and no ABMR groups. In the future, we envision the development of a more comprehensive and precise integrated algorithm that can further enhance risk prediction and ultimately improve renal transplant outcomes.

## Data Availability

The raw data supporting the conclusions of this article will be made available by the authors, without undue reservation.
