# Supplementary material for: An integrative algorithm combining HLA epitope registry, PIRCHE-T2, and PIRCHE-B outcomes to improve immunological risk stratification in kidney transplantation
Source: Front Immunol. 2026 Jan 16;16:1718506. doi: 10.3389/fimmu.2025.1718506 (PMC12855065; doi:10.3389/fimmu.2025.1718506)
Supplement: Supplementary file 1 [file DataSheet1.docx]

**Supplementary Figure 1.** ROC of Epregistry (A, D), PIRCHE-T2 (B, E), and PIRCHE-B (C, F) scores in the representative loci.

**
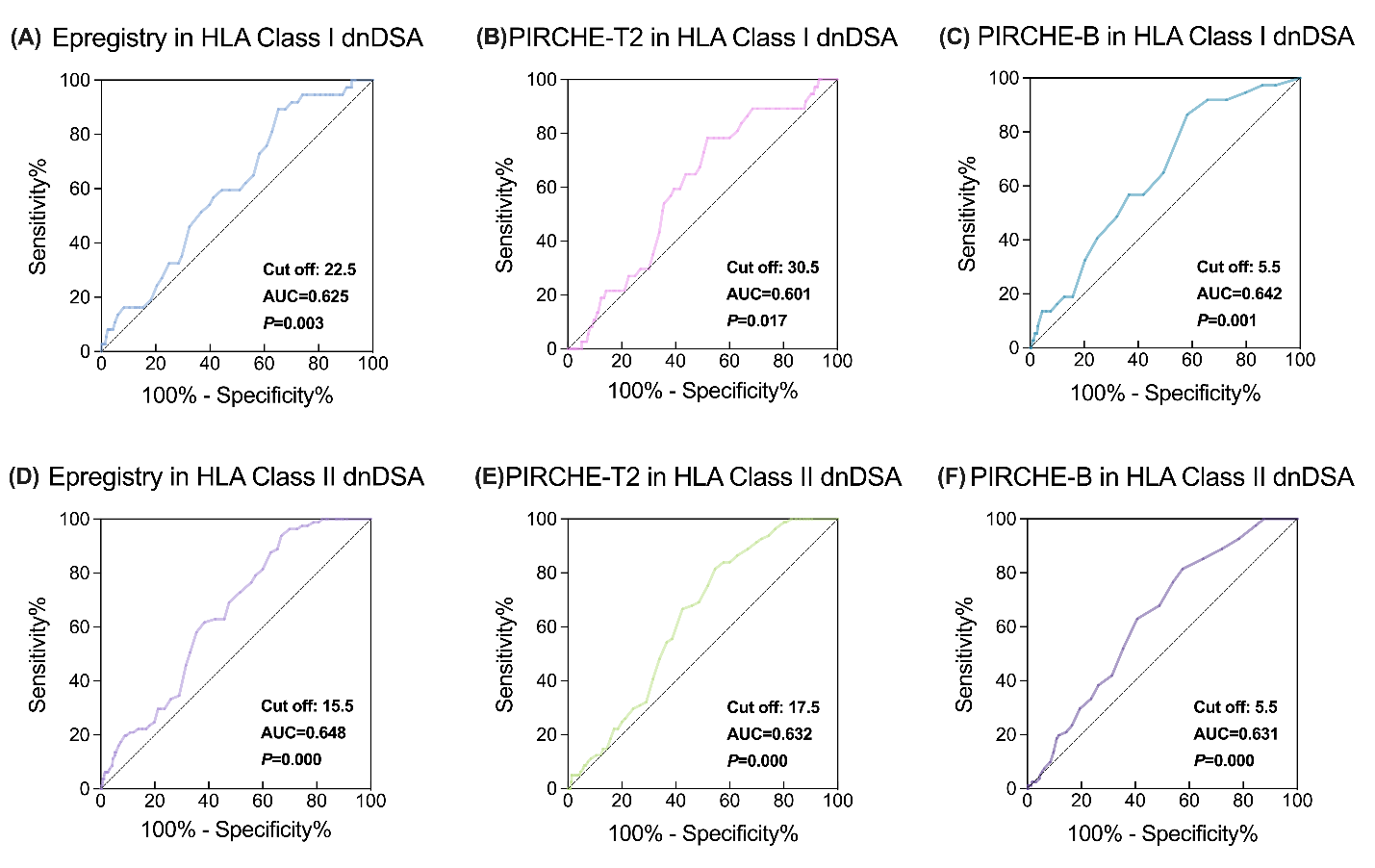
**

**Supplementary Table 1.** **Comparative characteristics of the HLA epitope prediction algorithms.**

| **Algorithm** | **Epregistry** | **PIRCHE-T2** | **PIRCHE-B** |
| --- | --- | --- | --- |
| **Immunogenicity Assessed** | Based on the inherent amino acid polymorphic structure of the HLA molecule. | Based on the potential for HLA-derived peptides to be processed, presented, and recognized by T-cell receptors. | Based on overall differences in atomic-level physical properties (solvent accessibility, protrusion) in 3D space. |
| **Input** | Donor/recipient HLA genotypes (amino acid sequences). | Donor/recipient HLA genotypes (requires HLA-I and HLA-II). | Donor/recipient HLA genotypes (requires high resolution for 3D modeling). |
| **Key Calculation & Threshold** | Epitope Matching: Queries a known Eplet database to check if the donor has "verified" or "verifiable" epitopes absent in the recipient. | Epitope Prediction & Counting: Uses a proprietary algorithm to predict the number of T-cell epitopes from donor HLA that can be presented by recipient HLA-II molecules. | 3D Physical Filtering: A site must pass both:  1. Snowflake: Sufficient solvent-accessible surface area.  2. Snowball: Sufficient local protrusion. |
| **Output** | Eplet Mismatch Count: A direct, easily interpretable count. | PIRCHE-T2 Score: A continuous value reflecting the potential for T-cell help. | PIRCHE-B Score: A count of mismatched amino acid positions that pass the dual physical filters. |

**Supplementary Table 2.** **Discriminative performance of the integrative algorithm before and after internal validation.**

| Loci analyzed | Original AUC | Split-sample validation AUC | Bootstrap Logistic | | | |
| --- | --- | --- | --- | --- | --- | --- |
|  |  |  | Groups | OR | 95% CI (Lower–Upper) | *P* - value |
| **HLA** Class I (A/B/C) | 0.687 | 0.687 | Intermediate V.S Low | 3.59 | -0.108 ~ 18.408 | 0.066 |
|  |  |  | High V.S Low | 9.56 | 1.160 ~ 19.273 | 0.004 |
| HLA Class II (DR/DQ) | 0.665 | 0.665 | Intermediate V.S Low | 5.73 | 0.705 ~ 19.185 | 0.012 |
|  |  |  | High V.S Low | 13.53 | 1.585 ~ 19.997 | <0.001 |

**Supplementary Figure 2.** **Kaplan–Meier curves for dnDSA-free and ABMR-free survival without truncation.**

**
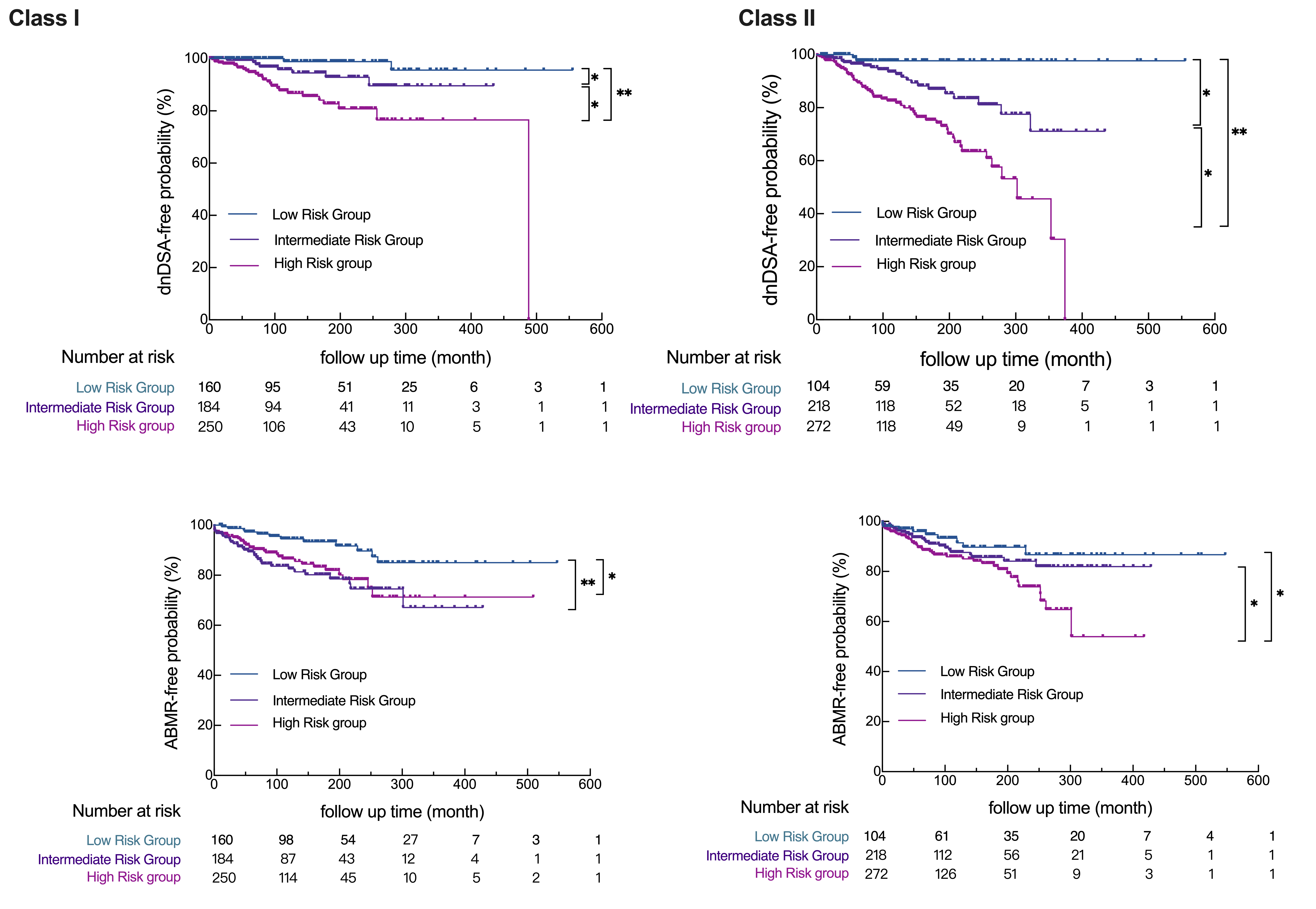
**

**Supplementary Figure 3. Comparison of tacrolimus trough levels between kidney transplant recipients with and without antibody-mediated rejection (AMR).**

**
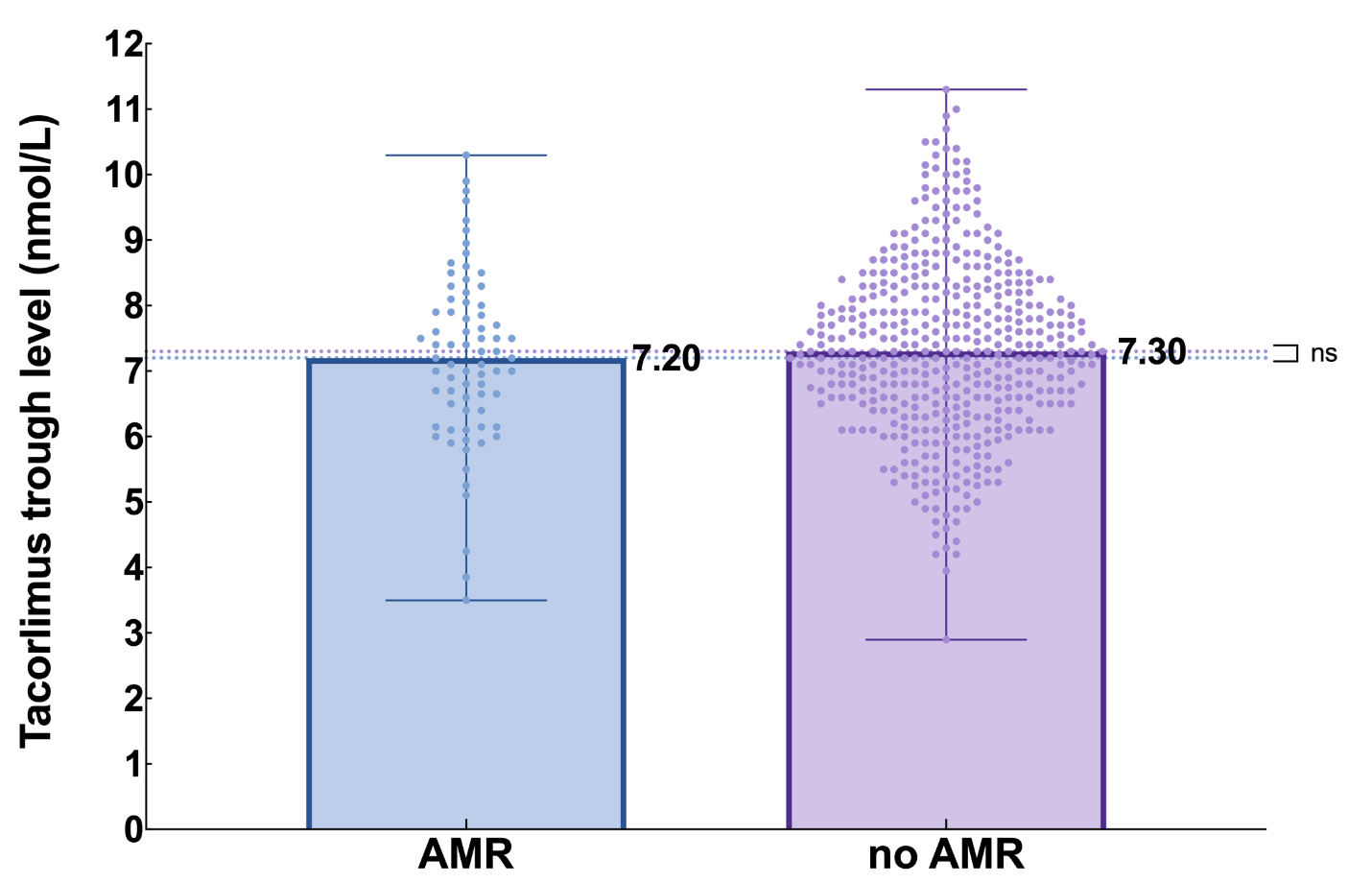
**

**Supplementary Table 3.** **Contingency Table for Phi Coefficient Analysis of dnDSA and ABMR**

|  | ABMR Present | ABMR Absent | Total |
| --- | --- | --- | --- |
| dnDSA Present | 48 | 56 | 104 |
| dnDSA Absent | 31 | 459 | 490 |
| Total | 79 | 515 | 594 |

The analysis was based on the following 2x2 contingency table from our cohort:

The formula for the Phi coefficient is: φ = (ad - bc) / √((a+b)(c+d)(a+c)(b+d))

Substituting the values: φ = (48×459 - 31×56) / √(79 × 515 × 104 × 490) ≈0.4457 ≈ 0.446

**Supplementary Figure 4.** **Percentage distribution of clinical events by risk groups based on the 3-algorithm.**

**
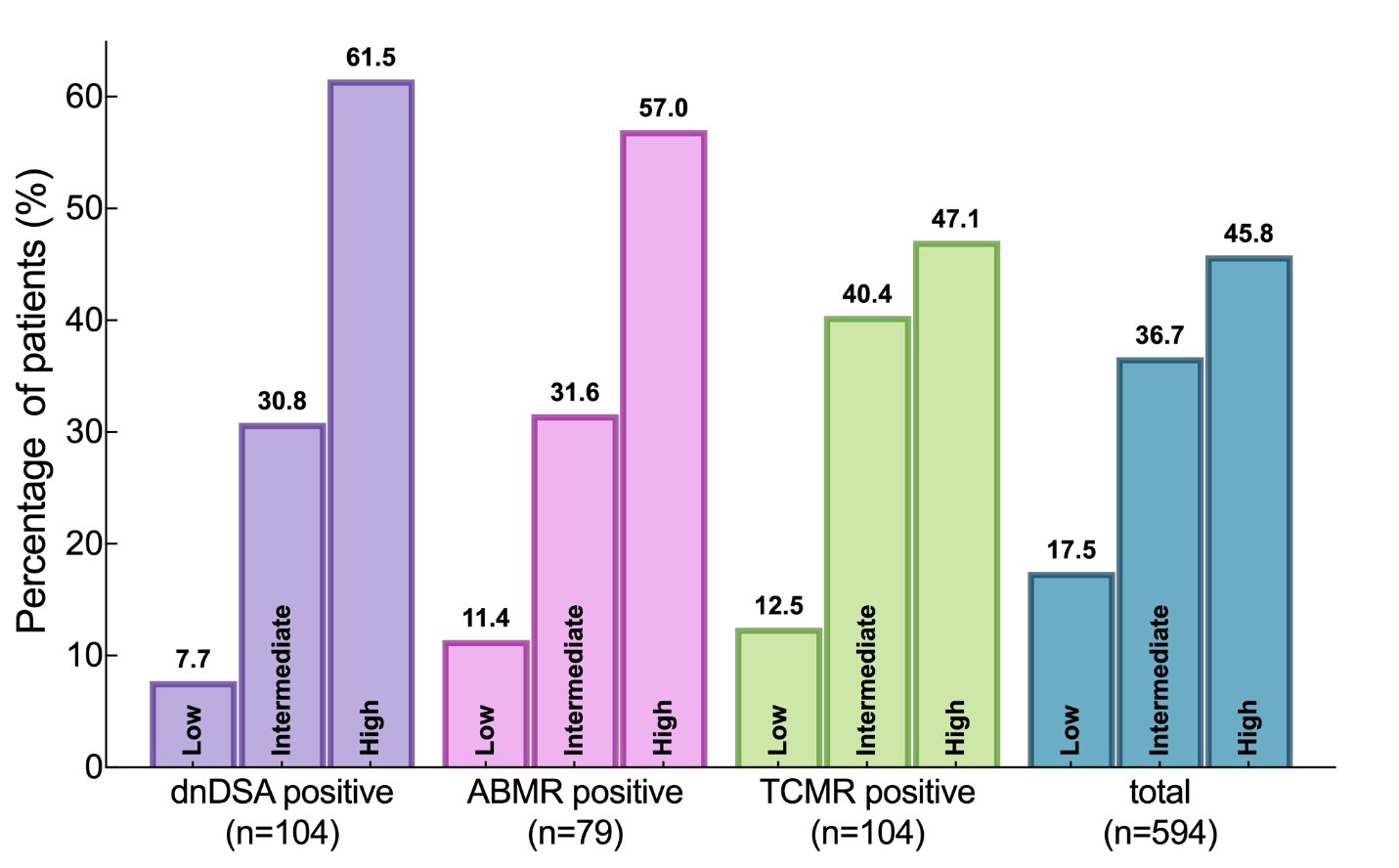
**

**Supplementary Table 4. Multivariable Cox proportional hazards regression analyses for dnDSA and ABMR endpoints**

| Endpoint | Number of patients | Number of events | Risk group | Risk group | HR (95% CI) | P |
| --- | --- | --- | --- | --- | --- | --- |
| dnDSA | 592 | 37(6.2%) | Class I | High vs Low | 15.11 (3.49–65.41) | <0.001 |
| dnDSA | 594 | 81(13.6%) | Class II | High vs Low | 20.55(4.93–85.60) | <0.001 |
| ABMR | 593 | 79(13.3%) | Class I | High vs Low | 2.21(1.11–4.38) | 0.024 |
| ABMR | 594 | 79 (13.3%) | Class II | High vs Low | 2.54(1.23–5.25) | 0.012 |

Separate Cox proportional hazards models were fitted for class I dnDSA, class II dnDSA, class I–associated ABMR, and class II–associated ABMR. All models were adjusted for recipient age, ethnicity, and donor type. Molecular mismatch risk classification was included as a categorical variable (low, intermediate, high), with the low-risk group serving as the reference category. HRs are reported with 95% confidence intervals.

**Supplementary Figure 5.** Epregistry HLA-C score (A), PIRCHE-T2 HLA-C score (B), PIRCHE-B HLA-B score (C), and PIRCHE-B HLA-C score (D) in TCMR and no TCMR patients. Box and Whisker plots represent the median (line in the middle of the box), 1st and 3rd quartiles (box), and 1.5x interquartile range (whisker). Outliers are depicted as dots outside the whiskers.

**
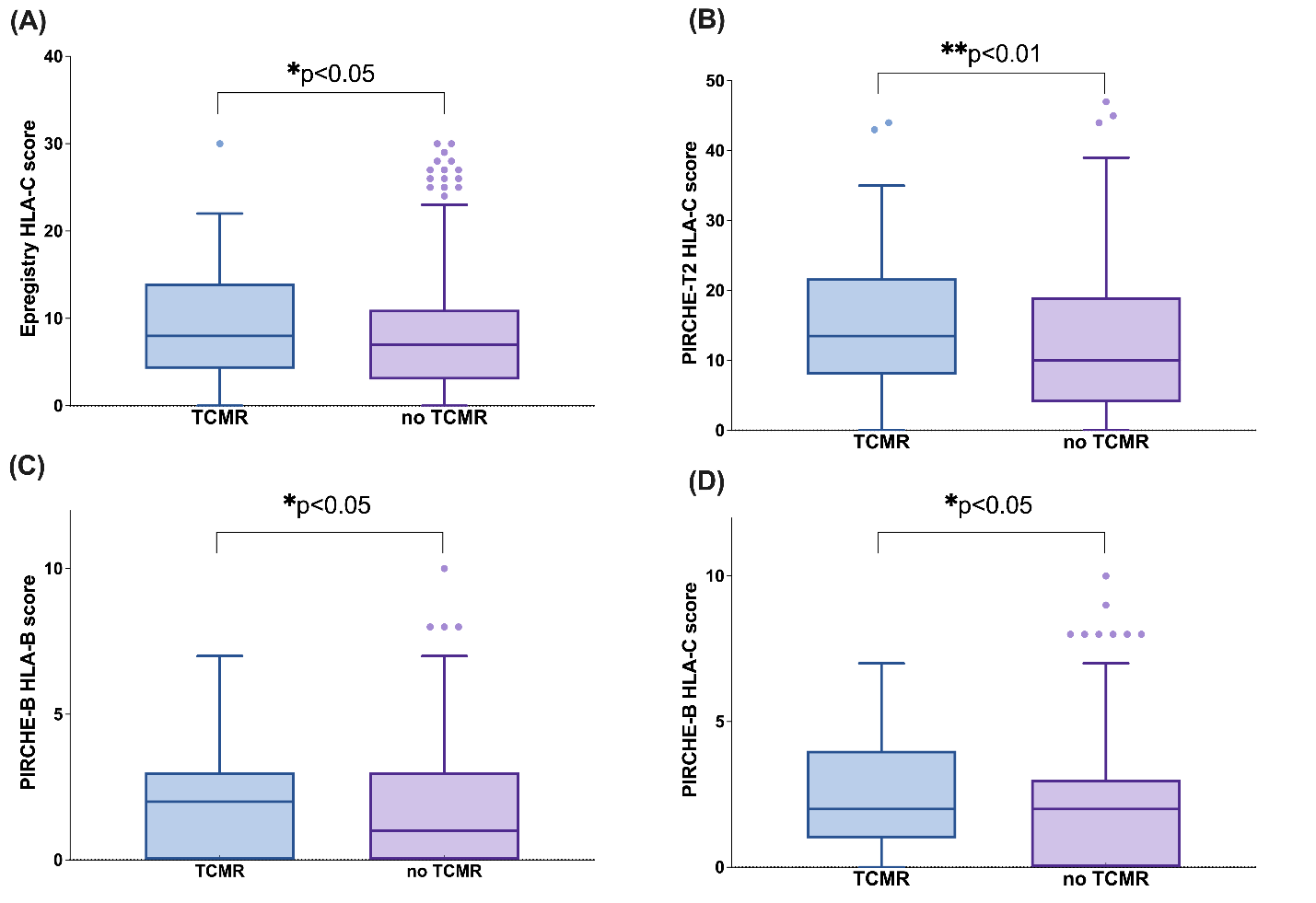
**
